# Systematic Review and Dose-Response Meta-Analysis on the Relationship between Different Gluten Doses and Risk of Coeliac Disease Relapse

**DOI:** 10.3390/nu15061390

**Published:** 2023-03-14

**Authors:** Mohammad Rostami-Nejad, Nastaran Asri, Meysam Olfatifar, Babak Khorsand, Hamidreza Houri, Kamran Rostami

**Affiliations:** 1 Gastroenterology and Liver Diseases Research Center, Research Institute for Gastroenterology and Liver Diseases, Shahid Beheshti University of Medical Sciences, Tehran 1985717411, Iran; 2 Gastroenterology and Hepatology Diseases Research Center, Qom University of Medical Sciences, Qom 3715614566, Iran; 3 Foodborne and Waterborne Diseases Research Center, Research Institute for Gastroenterology and Liver Diseases, Shahid Beheshti University of Medical Sciences, Tehran 1985717411, Iran; 4 Department of Gastroenterology, MidCentral DHB, Palmerston North 4442, New Zealand

**Keywords:** gluten proteins, coeliac disease, gluten-free diet, contamination, dose-response meta-analysis

## Abstract

Gluten proteins are known as immunological triggers for inflammation resulting in mucosal lesions in patients with coeliac disease (CD). Adherence to a strict gluten-free diet (GFD) is currently known as the only effective treatment for CD. In this study, we performed a systematic review and dose-response meta-analysis on data from previous studies to investigate the association between different gluten doses administered and the risk of CD relapse. Electronic databases were systematically searched to retrieve studies that investigated the response of CD patients to different amounts of gluten intake and evaluated the clinical, serologic, and/or histologic evidence to recognize disease relapse. Study-specific relative risks (RRs) were combined using a random effects model. A total of 440 identified published papers were screened, of which 7 records were selected following full-text reviewing and eligibility assessment for dose-response meta-analysis. According to our analysis, the risk of CD relapse is estimated to be 0.2% (RR: 1.002; 95% CI: 1.001 to 1.004) following the consumption of 6 mg gluten/day, which was increased to 7% (RR: 1.07; 95% CI: 1.03 to 1.10), 50% (RR: 1.50; 95% CI: 1.23 to 1.82), 80% (RR: 1.80; 95% CI: 1.36 to 2.38), and 100% (RR: 2.00; 95% CI: 1.43 to 2.78) by the daily intake of 150, 881, 1276, and 1505 mg gluten, respectively. Although good adherence to a GFD can adequately control CD-related symptoms, disease relapse might happen even with a very low dose of gluten, and the duration of exposure to gluten is also an important matter. The current literature has substantial limitations, such as relying on the data from just a few countries that were different in terms of the amount of gluten administered, the duration of the challenge, etc. Therefore, more randomized clinical trials using a standardized gluten challenge protocol are needed to confirm the findings of the present study.

## 1. Introduction

Coeliac disease (CD) is a lifelong systemic autoimmune disorder that develops by gluten protein consumption in genetically predisposed individuals [1,2]. In gluten-sensitive subjects, this protein promotes chronic inflammation of the intestinal epithelium, causing CD-related pathologic changes including crypt hyperplasia, villous shortening, and intraepithelial lymphocyte infiltration [3]. CD has heterogeneous and often vague clinical manifestations such as diarrhea, constipation, abdominal pain, fatigue, infertility, and dermatitis herpetiformis. The global prevalence of CD has been estimated to be approximately 1 to 3% in the general population and about 10%-20% in first-degree family members of CD patients [1,2,4]. CD development is strongly linked to the human leukocyte antigen (HLA) genes, located in the major histocompatibility complex (MHC) region on chromosome 6p21.3. HLA-DQ2 and/or -DQ8 alleles are the most important risk factors for this disorder [5,6,7,8]. CD related diagnostic testing includes evaluating serum levels of CD-specific antibodies, small-intestinal biopsy examination and HLA-DQ2 and HLA-DQ8 testing [9]. CD may manifest quite abruptly with acute features in a small proportion of patients, deferring the diagnosis and exposing these patients to possible life-threatening complications [10,11]. Gluten protein, as the storage protein fraction naturally found in some grains including wheat, barley, and rye, has a high content of proline (Pro) and glutamine (Gln) amino acids that are largely resistant to proteolysis by human digestive enzymes [12,13]. Partial digestion of gluten proteins generates immunomodulatory peptides which activate T-cell–mediated immune responses, followed by immunological inflammation and release of proinflammatory cytokines such as interferon-γ (IFN-γ) that result in mucosal injury in the small intestine of CD patients [14,15]. These histological findings are characteristic to CD but not specific and several disorders can be accompanied with comparable histopathological findings [9]. Gluten avoidance or adherence to a strict gluten-free diet (GFD) is currently known as the only effective treatment in remitting the symptoms of CD [16,17]. A GFD is an eating plan that contains naturally gluten-free foods (like meat, fish, vegetables, legumes, naturally gluten-free cereals, eggs, fruits, etc.) and manufactured products which are labeled as “gluten-free” [18]. Following a GFD in the long term is associated with improving the histological lesions, blood biochemistry, clinical manifestations and the risk of CD-related complications [19]. However, according to the Dutch Celiac Disease Association (NCV), there are many patients with persistent intestinal mucosal injury and clinical symptoms despite dietary gluten exclusion [1,20,21]. In fact, gluten is not always totally absent in processed foods and is often added to food products for improving their quality and stability [22]. Therefore, gluten-free (GF)-labeled commercial products are known as vehicles for a small amount of gluten that may result in a recurrence of CD-related symptoms [23,24]. Moreover, because of its adverse impacts on patients′ daily activities and the limits imposed on their social events, adherence to a strict GFD is not an easy task for the patients, which may lead to intentional or inadvertent exposure to gluten [25]. There is also evidence supporting the hypothesis that sensitivity to gluten and achieving tolerance to this protein are individual and depend on each particular patient, and there is a tremendous degree of variability between patients in terms of their responses to different amounts of gluten [24,26,27]. Hence, various studies and trials evaluated the effect of exposure to different doses of gluten on CD patients by assessing their clinical symptoms and histology [28]. Suffice it to say that excessive consumption of GF products may lead to gluten accumulation, which is accompanied by mucosal damage even if there is no CD-related typical symptom. In this regard, while Hischenhuber et al. [29] considered in their study the maximum allowed daily intake of gluten to be between 10 and 100 mg, other authors considered an average daily gluten intake of 34–36 mg to be safe for CD patients [30]. Unfortunately, some gluten challenge-based studies have serious limitations in their methodology. In the current study, we conducted a systematic review and dose-response meta-analysis on previous clinical trials to evaluate the association between different gluten doses and the risk of CD relapse.

## 2. Methods

### 2.1. Search Strategy, Data Retrieval, and Eligibility Criteria

This systematic review and meta-analysis was performed according to the Preferred Reporting Items for Systematic Review and Meta-Analyses (PRISMA) statement [31]. We implemented a dual-reviewer comprehensive literature search to find publications that investigated CD patients′ response to different amounts of gluten proteins. Accordingly, the following databases were included: Cochrane Library, MEDLINE (via PubMed), EMBASE, Scopus, and Web of Science. The following MeSH terms and keywords were used for the title and abstract screening: [“Gluten” OR “gluten-free” OR “gluten-free label” OR “gluten contamination” OR “gluten dose” OR “gluten dosage” OR “ gluten challenge” OR “gluten consumption” OR “gliadin” OR “prolamins” OR “hordein” OR “secalin” OR “glutelin”] AND [“celiac” OR “coeliac” OR “celiac disease” OR “CD” OR “CeD” OR “gluten enteropathy” OR “Gluten-Sensitive Enteropathy” OR “Nontropical Sprue” OR “Celiac Sprue”]. Search results were filtered to include clinical studies, clinical trials, controlled clinical trials, and randomized controlled trials restricted to studies published in English between January 1990 and December 2022. Our search strategy was supplemented by backward searches, in which the reference lists of the retrieved papers, as well as the relevant review articles, were screened to identify additional publications not detected by the initial search.

### 2.2. Study Selection, Quality Assessment, and Data Extraction

The titles and abstracts of the retrieved papers were screened independently by two reviewers (NA and HRH). Studies investigated the response of CD subjects to different amounts of gluten intake and evaluated the clinical, serologic, and/or histologic evidence to recognize if disease relapse was included. Data extraction was performed to retrieve the following items: the number of participants, gluten dosages and challenge duration, year of publication, the clinical signs of disease relapse, the country, and so on.

The quality assessment of included randomized controlled trials was appraised using the Cochrane Handbook for Systematic Reviews of Interventions, and the Newcastle–Ottawa Scale was used for the assessment of non-randomized studies [32]. Decisions on study eligibility and quality were made by two reviewers. Any discrepancies were resolved through discussion with a third reviewer.

### 2.3. Data Synthesis and Statistical Analysis

Two reviewers extracted datasets from the eligible studies independently. Any disagreement was resolved by discussion or by consulting a third researcher until a consensus was reached. Data synthesis and meta-analysis were performed using the “*dosresmeta*” and “*meta*” packages of R software (Version 4.5.1; R Foundation for Statistical Computing, Vienna, Austria). Relative risks (RRs) with 95% confidence intervals (CIs) were calculated for each study using a two-by-two table in the included studies. *I*^2^ value was used for assessing statistical heterogeneity between studies, which delineates the proportion of total variability across different studies, owing to heterogeneity rather than chance. Accordingly, *I*^2^ values of 25%, 50%, and 75% represented low, moderate, and high heterogeneity, respectively. Additionally, the Sidik–Jonkman method was used to estimate τ2 representing the study variance, and the Hartung–Knapp method to adjust test statistics, their CI, and, subsequently, the tests’ degrees of freedom. Finally, the publication bias was evaluated using the LFX index implemented in “*metasens*” R package. *p* value <0.05 was considered statistically significant.

The mutual relationship between gluten and relapse was assessed by dose-response meta-analysis using the “*dosresmeta*” package. Accordingly, we fitted linear and cubic spline models on our data.

## 3. Results

### 3.1. Characteristics of Included Studies

**Figure 1:**
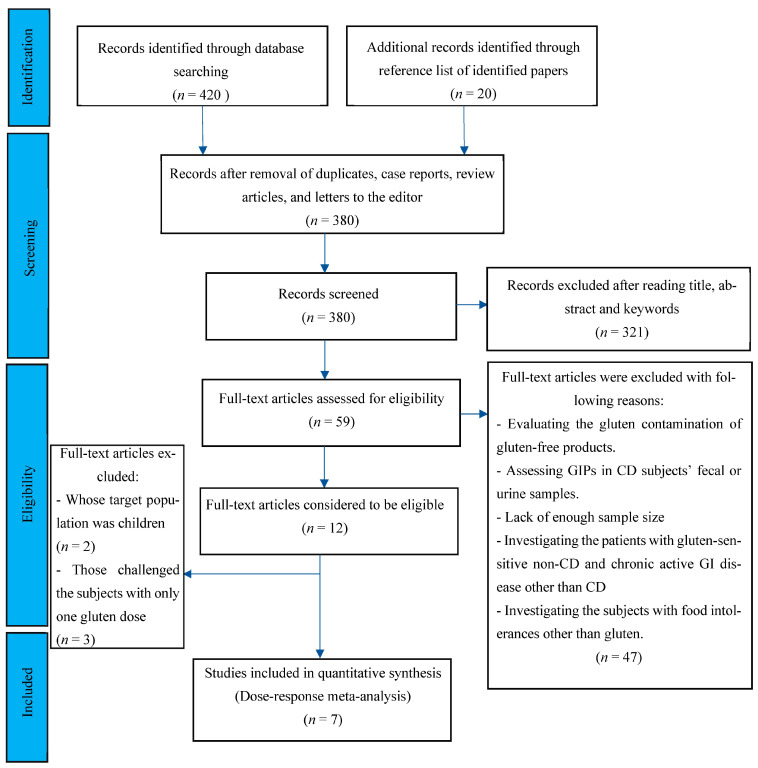
Flow chart showing the basis for selecting the studies used in the present analysis.

Following the databases’ search, 440 potentially eligible records were recognized in the initial screening published from 1990 to 2022. After the removal of duplicates, case reports, review articles, and letters to the editor, 380 records were considered for further screening of the titles and abstracts. Following screening titles, abstracts, and keywords, 59 records were included for full-text reviewing, eligible criteria evaluation, and quality assessment. After full-text, independent reviewing by 2 investigators, 47 papers were excluded for fitting any of the following categories: studies with the aim of evaluating the gluten contamination of gluten-free products; studies that measured gluten immunogenic peptides (GIPs) in fecal or urine samples of CD patients; studies without enough sample size; cases that evaluated the response of gluten-sensitive non-CD patients to gluten digestion; and studies including subjects with chronic active gastrointestinal disease other than CD or subjects with food intolerances other than to gluten. Two studies that enrolled pediatric cohorts and three papers with only one gluten dose challenge were excluded. The flow chart of the study selection process is outlined in Figure 1. Finally, seven studies met the eligibility criteria.

A total of 304 subjects were analyzed, and the challenge duration ranged from 7 days to 90 days. Table 1 summarizes the details of the extracted data from the eligible studies in the meta-analysis.

**Figure 2:**
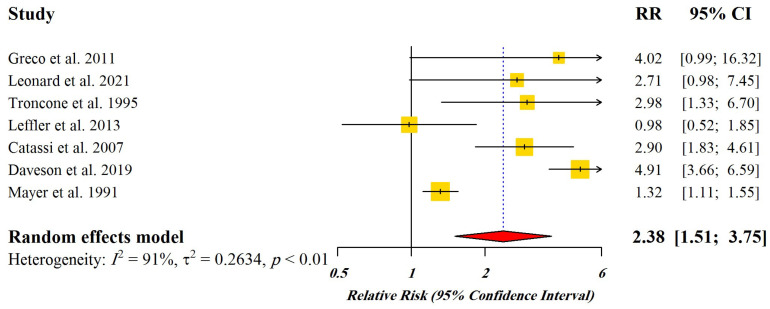
Forest plot of pooled relative risks (RR) with 95% CI for included studies [4,21,33,34,35,36,37]. Yellow Squares: Point estimate of risk ratio, Red Diamond: Total estimate of risk ratio.

Figure 2 displays the Forest plot indicating pooled relative risks (RRs) with 95% CI for the studies enrolled for dose-response meta-analysis.

### 3.2. Dose-Response Meta-Analysis

**Figure 3:**
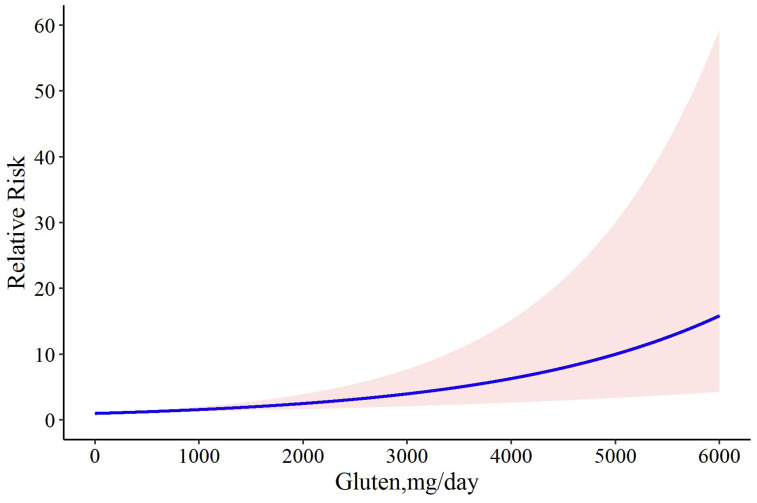
The dose-response meta-analysis of gluten dose and risk of CD relapse. The solid curve and the shadow area indicate the relative risk with a corresponding 95% confidence interval.

In a meta-analysis of all studies, we found a linear dose-response relationship between the levels of gluten consumption and relapse risk (Chi2 = 16.91, *p*-value = 0.001) (Figure 3), in which the coefficient of the model was 0.0005. In addition to the linear model, we also tried to fit a cubic spline model with 3 knots of 0.25, 0.50, and 0.75 to our data. The results of the goodness-of-fit test for the linear model and the cubic spline model were D = 18.182, *p*-value = 0.077 versus D = 36.142, *p*-value = 0.001, respectively; hence, the latter model failed to explain the intrinsic association between gluten and relapse in our study. Moreover, the R^2^ values of the 2 models were 0.482 and 0.124 for the linear and cubic spline models, respectively. These results confirm the superiority of the linear model in our study over the cubic spline model. According to our results, the risk of CD relapse is estimated to be 0.2% (RR: 1.002; 95% CI: 1.001 to 1.004) following the consumption of 6 mg gluten/day, which was increased to 0.4% (RR: 1.004; 95% CI: 1.002 to 1.006), 1.8% (RR: 1.018; 95% CI: 1.009 to 1.02), 7% (RR: 1.07; 95% CI: 1.03 to 1.10), 50% (RR: 1.50; 95% CI: 1.23 to 1.82), 80% (RR: 1.80; 95% CI: 1.36 to 2.38), and 100% (RR: 2.00; 95% CI: 1.43 to 2.78) by the daily intake of 10, 40, 150, 881, 1276, and 1505 mg gluten, respectively (Figure 3).

### 3.3. Sensitivity Analysis

**Figure 4:**
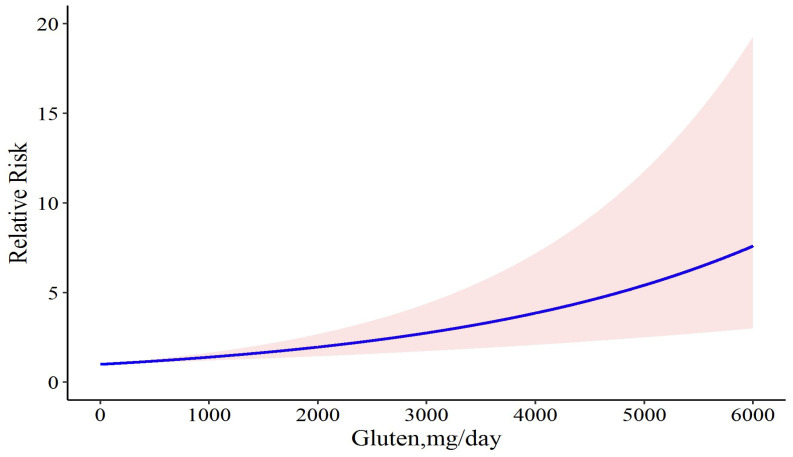
The dose-response meta-analysis of gluten dose and risk of CD relapse following sensitivity analysis. The solid curve and the shadow area indicate the relative risk with a corresponding 95% confidence interval.

For a better insight into the results, we tried to determine whether the exclusion of studies with two doses of gluten would improve the results. Accordingly, we first excluded the study by Daveson et al., in which case there was a similar association between gluten and relapse risk (Chi2 = 18.30, *p*-value = 0.001), but the model coefficient was reduced to 0.0003. Heterogeneity was not significant and decreased (Q = 9.99, *p*-value = 0.075, and *I*^2^ = 50.0%). This model had less deviance (D = 16.50 vs. D = 18.182) and better R^2^ (0.526 vs. 0.482) than the full model. This result can show that removing the study by Daveson et al. can lead to providing a better model. Likewise, after removing the study by Leonard et al., the model coefficient was again equal to 0.0003; the heterogeneity was not significant; and the model R^2^ was 0.468. This model was not prioritized over the previous and complete models. Likewise, after removing the study by Catassi et al., the coefficient of the model increased to 0.0004, and the heterogeneity was significant. In addition, the model R^2^ was 0.446 and the deviance test was D = 16.07, *p*-value = 0.041, which can show that the model does not have the necessary goodness-of-fit. Furthermore, in all aforementioned situations, the linear model has better fitness on our data than the cubic spline model. Therefore, we concluded that the model that does not have the study by Daveson et al. was the ideal model for our study. We performed our dose-response meta-analysis of gluten dose and risk of CD relapse again and found that daily consumption of 6 mg gluten resulted in 0.2% disease relapse (RR: 1.002; 95% CI: 1.001 to 1.002), and that it was increased to 0.3% (RR: 1.003; 95% CI: 1.001 to 1.004), 1.3% (RR: 1.013; 95% CI: 1.007 to 1.01), 5% (RR: 1.05; 95% CI: 1.02 to 1.07), 50% (RR: 1.50; 95% CI: 1.24 to 1.8), 80% (RR: 1.80; 95% CI: 1.37 to 2.35), and 100% (RR: 2.00; 95% CI: 1.45 to 2.74) following 10, 40, 150, 1200, 1739, and 2050 mg gluten intake per day, respectively (Figure 4).

### 3.4. Correlation between CD Relapse and Studies’ Features 

**Figure 5:**
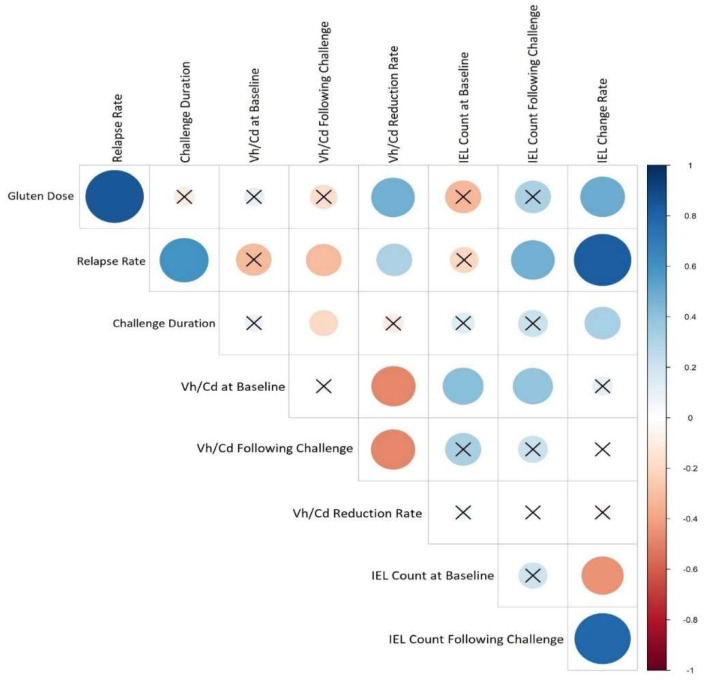
Heat map corresponding to the correlation between CD relapse and studies’ features. Each lattice color represents the intensity of the correlation, with blue representing a positive correlation coefficient and red representing a negative one. Crossed circles represent a non-significant correlation. Large circles indicate a stronger correlation between the row and the column variables.

Figure 5 presented the correlations between the study variables, the most important of which is that the disease relapse has a significant positive correlation with the duration of the challenge (*p* value = 0, r = 0.78) in addition to the gluten dose (*p* value = 0, r = 0.82). In more detail, gluten dosage was positively correlated with villous height/crypt depth (Vh/Cd) reduction rate (*p* value = 0.03, r = 0.47), and challenge duration had a negative correlation with Vh/Cd ratio following the challenge (*p* value = 0.04, r= −0.15). The intraepithelial lymphocyte (IEL) change rate was positively correlated with both the gluten dose (*p* value = 0.02, r = 0.49) and challenge duration (*p* value = 0.04, r = 0.31).

### 3.5. Publication Bias

**Figure 6:**
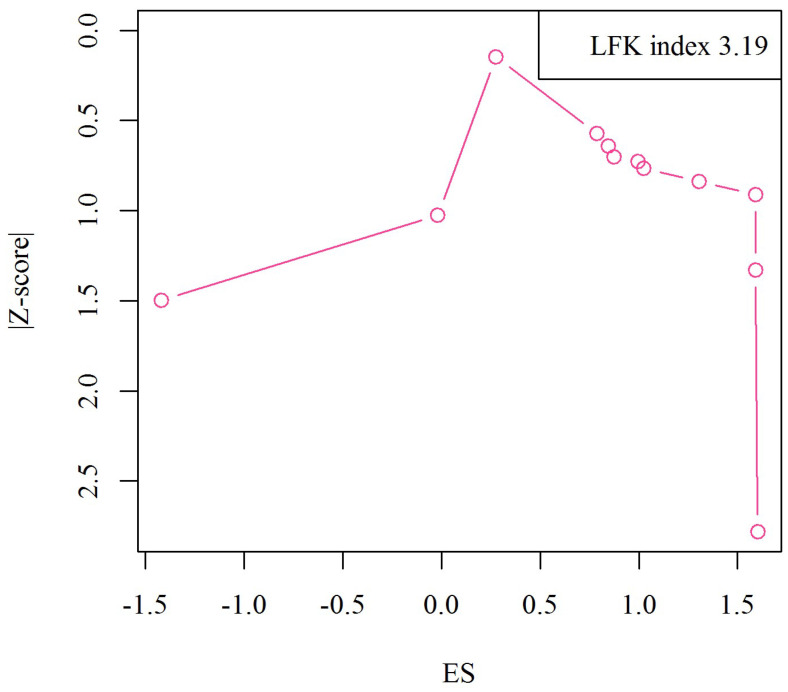
Doi plot analysis and LFK index of publication bias.

As shown in the Doi plot (Figure 6), we observed significant publication bias in the included studies (LFX index: 3.19, major asymmetry).

## 4. Discussion

Gluten is a protein naturally found in certain cereal grains that can be consumed as much as 7.5 g/day by women and 10 g/day by men on a typical diet [38]. The Western diet is also reported to contain about 5–15 g of gluten per day [39] and its consumption during infancy is high in Sweden [40]. 

As lifelong strict adherence to a GFD is considered the only accepted treatment for CD patients, leading to the healing of the duodenal mucosa and the resolution of the patients’ symptoms, gluten-free product labeling plays an important role in determining the food choices for CD patients [41,42]. Since commercially available GF products may be contaminated by tiny amounts of gluten proteins during their milling, storage, manipulation, and so on, achieving a strict GFD without any gluten content is almost impossible; hence, many CD patients following GFD still experience active disease-related symptoms [33,43]. In fact, although the understanding of CD pathophysiology, diagnosis, and clinical manifestations has increased over time, limited data are available regarding the potential toxicity of different amounts of gluten for CD patients. Paying attention to this problem is clinically important as persistent intestinal mucosal injury can be accompanied by increased risks of several CD-related complications like osteoporosis [21]. In these types of studies, considering gluten responsiveness as a defining feature of CD, clinical researchers try to evaluate the patients’ response to different amounts of gluten, and their results can be considered vital for both patient care and clinical trial designs. However, due to a lack of standardization of important factors such as time and gluten dose, the available data are different from each other in their objectives and designs [44]. In the current study, we tried to conduct a systematic review and dose-response meta-analysis on previously published data to explore the potential of different amounts of gluten in the induction of disease relapse in patients with CD, which can be an important step in advancing CD management.

The main finding in the current study suggested a dose-dependent association between the amount of consumed gluten and CD relapse. According to our results, an intake of 6 mg/day of gluten is accompanied by a 0.2% chance of disease relapse, which is increased to 1.8% by consumption of 40 mg of gluten per day. With the increase in gluten consumption, the chance of relapse also increased and reached 50% in the amount of 881 mg and 100% in the amount of 1.5 g of gluten/day. In other words, the dose-response curve showed a positive correlation between gluten dose and the risk of CD relapse, characterized by an initial slight increase in risk at the lower dosage followed by a steeper rise at over 1.5 g/day. It is noteworthy that challenge duration also showed a positive correlation with CD relapse, which shows the importance of paying attention to the duration of exposure to gluten as much as the amount of gluten consumed. For ethical reasons, gluten-response evaluating studies usually limit the duration of their micro challenge to short times (usually less than 3 months), but in most cases, the mucosal deteriorating effects of gluten manifest following a longer gluten challenge [33]. In our dose-response meta-analysis, the linear regression showed a significantly better fit than the cubic spline regression, indicating a linear association of gluten consumption with disease relapse. It should also be considered that it is not only the quantity of gluten that is effective in patients’ reactions; the quality of the protein which is influenced by cereal variety and food processing methods might affect the severity of the reaction as well [33]. Furthermore, epidemiological criteria, as well as dietary habits, could affect the disease relapse [33]. 

The present study had some limitations. First, the included studies implemented different approaches to evaluate disease relapse in response to gluten challenges. These approaches included evaluating patient-reported symptoms that are subjective; assessing serological biomarkers that are not sensitive enough; assessing histological damages such as Vh/Cd ratio and IEL count that allow investigators to evaluate villous blunting and lymphocyte infiltration but require assessment by a skilled pathologist [33,44]. The studies also differ from each other in terms of sample size, the amount of gluten administered, duration of the challenge, etc. In addition, histological recovery is not only often unrelated to clinical recovery, but it can also be slow and incomplete in a substantial number of patients, which would make interpretation based on histology less reliable [45]. Moreover, the findings in the present study are just from studies assessing the adult population with CD (Parents usually are reluctant to expose their child to gluten to avoid CD-related symptoms reocurance). Finally, the data from such clinical trials are influenced by other factors such as ethnicity and geographical parameters. For instance, a major number of clinical trials on CD relapse have been conducted in Italy. Therefore, our reports should be verified using large-scale prospective controlled studies involving a multicenter approach with extensive cohorts.

## 5. Conclusions

Although good adherence to a GFD can adequately control CD-related symptoms, disease relapse might happen from even a very low dose of gluten. The duration of exposure to gluten can also be an important factor in the frequency and severity of disease relapse. Remarkably, performing randomized clinical trials in various countries using a standardized gluten challenge protocol and morphometric analysis of the small-intestinal mucosa using quantitative histology (Vh/Cd index and the IEL count) might be effective in reaching more clarity and definitive results. More importantly, it is crucial to screen and monitor the amount of gluten received from all kinds of foods consumed during the day by CD patients. We would like to emphasize the importance of re-evaluating GFD under the guidance of a dietician for CD patients. It should also be considered that the current conclusion may be modified as new information develops through further studies.

## Figures and Tables

**Table 1:** Characteristics of the included studies.

Study	Study Design	Geographic Location	Interventions	Number of Participants	Challenge Duration	Relapse Assessment Method	Relative Risk (RR)	Confidence Interval 95%
Daveson et al., 2019 [21]	Randomized, double-blind, sham-controlled gluten challenge trial	United States, New Zealand, Australia	Placebo	36	14 days	Clinical symptoms	1 (Ref.)	-
			6000 mg gluten/day	36			4.9127	3.6609–6.5925
Leonard et al., 2021 [4]	Randomized, double-blind, two-dose gluten challenge trial	Boston, USA	3000 mg gluten/day	7	14 days	Pathological changes	1 (Ref.)	-
			10,000 mg gluten/day	7			2.7081	0.9842–7.451
Catassi et al., 2007 [33]	Multi-center, randomized, double-blind, placebo-controlled, trial	Italy	10 mg gluten/day	13	90 days	Pathological changes	2.3307	1.2271–4.4271
			50 mg gluten/day	13			3.6888	1.8887–7.205
			Placebo	13			1 (Ref.)	-
Leffler et al., 2013 [34]	Randomized, double-blind, two-dose Gluten challenge trial	Israel	3040 mg gluten/day	10	14 days	Pathological changes	1 (Ref.)	-
			7500 mg gluten/day	10			0.9808	0.521–1.8464
Greco et al., 2011 [35]	Randomized, double-blind clinical trial	Italy	16,025 mg gluten/day	6	60 days	Clinical complications (CD related antibodies and biopsy findings)	2.3978	0.1784–32.2328
			496 mg gluten/day	2			4.9628	0.9379–26.2608
			1.6 mg gluten/day	5			1 (Ref.)	-
Troncone et al., 1995 [36]	Randomized, double-blind clinical trial	Italy	<500 mg gluten/day	6	7 days	Pathological changes	2.1972	0.5768–8.3694
			500–2000 mg gluten/day	6			2.7850	0.7224–10.7375
			>2000 mg gluten/day	7			4.9052	1.0453–23.0197
			Control (strict GFD)	4			1 (Ref.)	-
Mayer et al., 1991 [37]	Randomized, double-blind clinical trial	Italy	60–2000 mg gluten/day	14	30 days	Pathological changes	−0.2418	0.1732–0.3376
			˃2000 mg gluten/day	29			1.3163	1.1147–1.5543
			Control (0 mg gluten/day)	80			1 (Ref.)	-

## Data Availability

Not Applicable to this study.
